# Unveiling Gene Interactions in Alzheimer’s Disease by Integrating Genetic and Epigenetic Data with a Network-Based Approach

**DOI:** 10.3390/epigenomes8020014

**Published:** 2024-04-01

**Authors:** Keith L. Sanders, Astrid M. Manuel, Andi Liu, Boyan Leng, Xiangning Chen, Zhongming Zhao

**Affiliations:** 1 Center for Precision Health, McWilliams School of Biomedical Informatics, Houston, TX 77030, USA; keith.l.sanders@uth.tmc.edu (K.L.S.); astrid.m.manuel@uth.tmc.edu (A.M.M.); andi.liu@uth.tmc.edu (A.L.); xiangning.chen@uth.tmc.edu (X.C.); 2 Department of Epidemiology, Human Genetics and Environmental Sciences, School of Public Health, Houston, TX 77030, USA

**Keywords:** epigenetics, DNA methylation, genome-wide association studies (GWAS), protein–protein interaction, network analysis

## Abstract

Alzheimer’s Disease (AD) is a complex disease and the leading cause of dementia in older people. We aimed to uncover aspects of AD’s pathogenesis that may contribute to drug repurposing efforts by integrating DNA methylation and genetic data. Implementing the network-based tool, a dense module search of genome-wide association studies (dmGWAS), we integrated a large-scale GWAS dataset with DNA methylation data to identify gene network modules associated with AD. Our analysis yielded 286 significant gene network modules. Notably, the foremost module included the BIN1 gene, showing the largest GWAS signal, and the GNAS gene, the most significantly hypermethylated. We conducted Web-based Cell-type-Specific Enrichment Analysis (WebCSEA) on genes within the top 10% of dmGWAS modules, highlighting monocyte as the most significant cell type (*p* < 5 × 10^−12^). Functional enrichment analysis revealed Gene Ontology Biological Process terms relevant to AD pathology (adjusted *p* < 0.05). Additionally, drug target enrichment identified five FDA-approved targets (*p*-value = 0.03) for further research. In summary, dmGWAS integration of genetic and epigenetic signals unveiled new gene interactions related to AD, offering promising avenues for future studies.

## 1. Introduction

Alzheimer’s Disease (AD) is a progressive neurodegenerative disease that causes brain atrophy [1]. AD is the primary cause of dementia, which includes symptoms of cognitive impairment, difficulty in memory recall, behavioral instability, and inability to perform daily tasks [2]. As the disease progresses, individuals at the age of 65 and older are particularly vulnerable to experiencing an escalation in symptom severity, reflecting the prevalence of AD in this age group [3]. AD and other dementias affect approximately 55 million individuals worldwide [4]. The neuropathological characteristics of AD involve extracellular deposits of β-amyloid and intraneuronal accumulation of neurofibrillary tangles [1]. Although AD has become easier to diagnose than before, there still exists a limited understanding of how the disease occurs and what factors contribute to the progression of the disease. It remains a challenge for early detection and prevention. As the global population continues to age, the prevalence of AD has increased over time, making it imperative to prioritize the understanding of the underlying causes of AD [5].

AD is designated as a complex disease due to its association with several genetic, environmental, and lifestyle factors, which are not well understood and are challenging to address [6]. Extensive research has focused on various genetic elements associated with AD. For example, different isoforms of apolipoprotein E (APOE) have been researched for their association with AD, with each isoform exhibiting different associations with the risk of developing AD [7]. Genetic studies have identified mutations in genes *APP*, *PSEN1*, and *PSEN2* as causative for autosomal dominant forms of AD, providing a foundation for identifying additional genetic risk loci [8]. During the past decade, several large-scale, population-based genome-wide association studies (GWAS) have been conducted to investigate AD, uncovering the cumulative effect of thousands of single-nucleotide polymorphisms (SNPs) on the pathogenesis of AD [9,10,11,12,13]. Previous research has identified 75 genetic risk loci associated with AD predisposition [13]. However, the functional mechanisms by which these variants exert their combined effects on AD pathogenesis at the transcriptomic and epigenetic levels remain unclear.

Epigenetics explores how gene expression is regulated and modified through mechanisms that do not involve changes to the underlying DNA sequence [14]. DNA methylation is a well-studied epigenetic mechanism involving methylation of cytosine bases of the dinucleotide CpG [14,15,16]. It is a critical process in epigenetic regulation, offering insights into genetic susceptibility and environmental influences. Consequently, it may significantly contribute to the intricate gene regulation mechanisms defining AD’s molecular basis. Moreover, DNA methylation levels increase with the aging process of individuals, marking this epigenetic modification for consideration in age-rated diseases [17]. Studies have indicated that epigenetic aging observed in elderly individuals, measured through DNA methylation patterns, is discrete from telomere attrition and cellular senescence. This distinction suggests that DNA methylation could significantly influence cellular processes and metabolism as we age [18,19]. Moreover, a study by Buckland et al. demonstrated that DNA methylation patterns at specific sites could be utilized in regression modeling as a predictor variable for age [20]. As such, investigating epigenetic factors may be a substantial leap forward in improving our understanding of AD development risks.

Due to the heterogeneous nature of AD and our limited understanding of gene interactions associated with the disease, we utilized our in-house tool, the dense module search of genome-wide association studies (dmGWAS), to identify gene networks associated with AD. dmGWAS is a robust network-based method designed to identify the complex interplay between disease-associated genes by integrating GWAS data with a reference network such as the human protein–protein interaction (PPI) network [21]. The tool dmGWAS was previously applied to integrate genetic and epigenetic data [22]. The current study incorporated DNA methylation data from the AD post-mortem brain as the epigenetic element within this framework. By integrating DNA methylation data, dmGWAS bridged the gap between genetic predisposition and environmental effects, enhancing our understanding of AD’s molecular underpinnings. This approach facilitated the discovery of AD-related gene interactions, yielding new insights into the disease’s functional pathology and supporting the development of targeted therapeutic strategies.

## 2. Results

**Figure 1:**
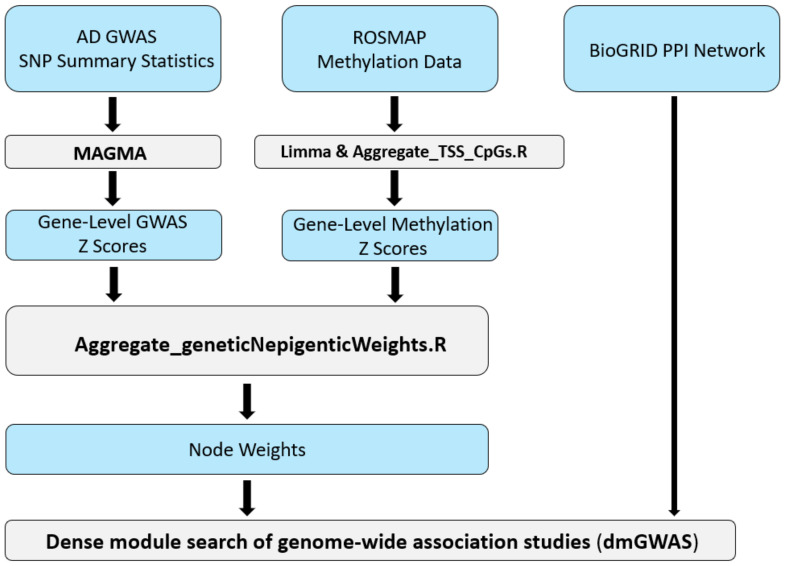
Workflow for implementation of the network-based method, dmGWAS, for integrative GWAS and methylation study of AD. This pipeline illustrates the integrative workflow for preprocessing and incorporating each dataset into the dense module search of genome-wide association studies (dmGWAS) tool. Both AD GWAS and CpG-level DNA methylation data are integrated using a combination of publicly available bioinformatics tools and custom R scripts.

Our AD dmGWAS model was constructed using publicly available data, including one of the largest AD GWAS datasets, the DNA methylation data from the AD post-mortem brain, and the human PPI reference dataset [23,24,25]. Figure 1 depicts the full pipeline of our integrative systems biology approach. This pipeline details how the multi-omics data were transformed and integrated using the dmGWAS tool.

### 2.1. Constructing the AD-Associated Top Network Modules

Summary statistics were derived from the GWAS in Wightman et al. [23], which included a total of 398,058 subjects of European descent. Of these individuals, 39,918 were diagnosed with AD and attributed to the cases group (Table 1). The DNA methylation dataset was downloaded from The Religious Orders Study and the Memory and Aging Project (ROSMAP) and includes comprehensive omics data from post-mortem human brain samples. We utilized DNA methylation and clinical demographic profiles from 549 participants, including 325 cases and 224 controls (Table 1) [24]. The PPI reference network utilized in our study was retrieved from the Biological General Repository for Interactions Dataset (BioGRID), a comprehensive database of curated genetic and protein interactions [25]. We preprocessed the PPI dataset by excluding non-human and redundant data, resulting in a refined dataset comprising 19,087 genes and 536,844 unique PPIs. The dmGWAS tools integrate these datasets and identify highly enriched modules with genetic and epigenetic signals (see Section 5 for details on module score calculation).

### 2.2. AD-Associated Top Modules from dmGWAS Analysis

The dmGWAS analysis identified 286 network modules that had significantly enriched module scores. The module score (Z_m_) is calculated to evaluate the density of low *p*-value genes within a module by aggregating the individual scores of genes (Z_i_) within the module, adjusted for the number of genes (k) in the module (see Section 5). We ranked the module based on normalized scores derived from permutation testing, which assesses the significance of the module scores. The top module of dmGWAS was visualized using the Cytoscape tool [26]. For visualization, we encoded the GWAS association signals as the node weight, reflected by the color. The node weight represents a Z score for each gene based on GWAS signals within the respective gene region (see Section 5). The node fill color gradient begins at 1.96, representing statistical significance (*p* < 0.05), and increases as the GWAS signal increases. The methylation signal was encoded wherein the methylation Z score exceeds 1.96, indicating statistically significant hypermethylation. Conversely, significant hypomethylation was encoded with a methylation Z score below −1.96. The distributions of gene-level GWAS Z score weights and gene-level methylation Z score weights are in the Supplementary File (SF1).

**Figure 2:**
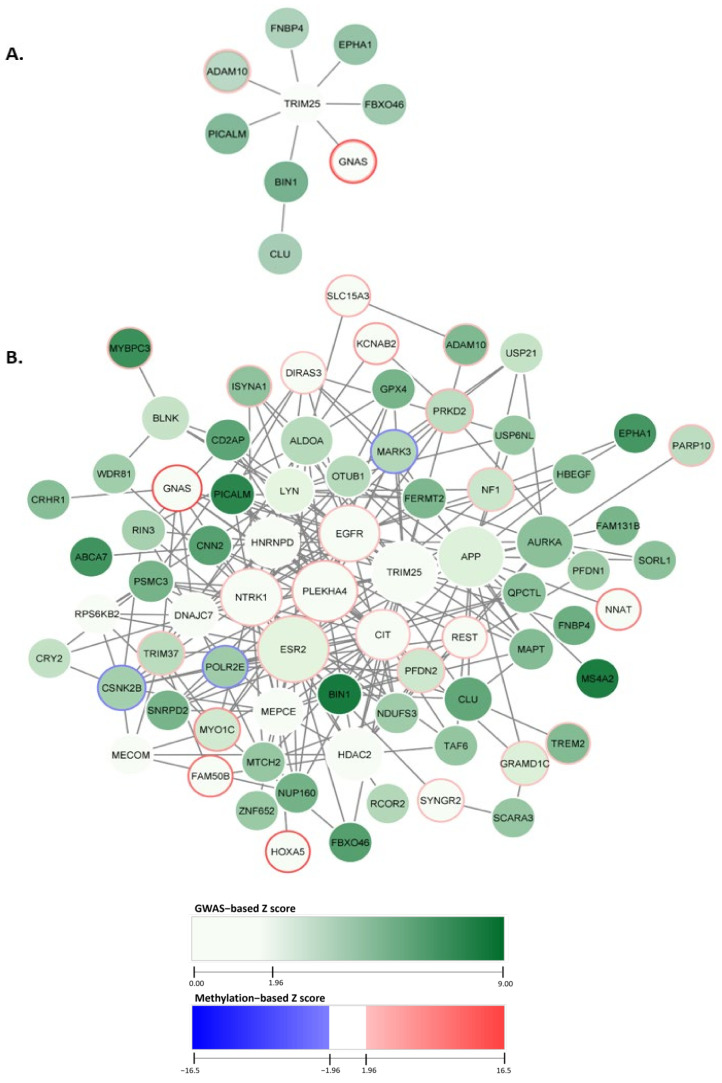
dmGWAS network module discovery revealed AD gene sets with enriched genetic and methylation signals. In the figure, each node represents a gene, and an edge connects nodes symbolizing interactions within the PPI network. Node color intensity reflects GWAS−based Z scores. Border colors indicate methylation intensity and directionality based on methylation Z scores. Here, blue depicts hypermethylation, and red depicts hypomethylation. (**A**) The top module indicated by dmGWAS is displayed. (**B**) The genes found within the top 10% of dmGWAS modules are displayed. The legend encoding remains the same as Figure 2A; however, node size represents centrality betweenness measurement, wherein larger nodes indicate higher betweenness.

We designated the top module, comprised of nine genes, with a normalized module score (Z_m_) of 19.11, as seen in Figure 2A. The top module’s central node was *TRIM25,* connected to seven genes within the top module. Additionally, *CLU* was included in the top module solely through its interaction with the gene *BIN1*. The gene identified with the largest GWAS node weight was *BIN1*, weighing 8.31. The gene *GNAS* indicated the highest methylation value of 16.47.

We empirically selected the top 10% of these modules (n = 29) (with the highest enrichment scores) to evaluate our findings. The top 10% of dmGWAS modules are shown in Figure 2B. This network of merged network modules was composed of 74 genes. Node size represented the measurement of betweenness centrality (see Section 5). Of the 74 genes included in this network, 18 exhibited statistically significant hypermethylation. This network indicated the genes *CSNK2B*, *MARK3*, and *POLR2E* as hypomethylated. A complete list of the node weights can be found in the Supplementary Files (Table S1).

### 2.3. Enrichment Analyses of Top 10% Modules Suggested AD-Related Cell Type and Pathways

Enrichment analyses were performed for the biological interpretation of the genes in the dmGWAS modules. For this purpose, we investigated the top 10% modules (n = 29) identified by dmGWAS, which included 74 genes.

**Figure 3:**
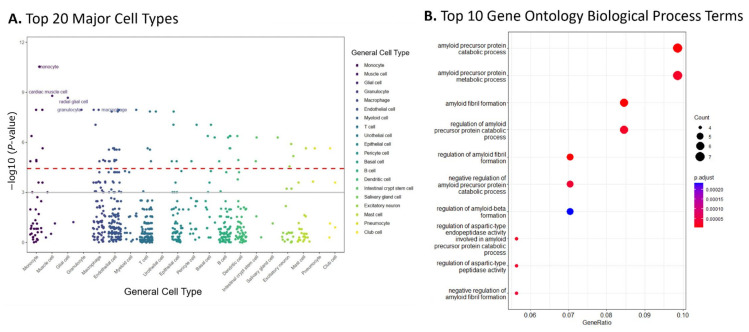
Enrichment analysis of the top 10% of modules suggested AD relevant cell types and pathways. (**A**) Results from WebCSEA data showcasing the top 20 major cell types enriched in the genes of the top 10% of dmGWAS modules, displaying monocytes, muscle, glial, and macrophage cell types. The red dashed lines represent a significant threshold (*p* = 3.69 × 10^−5^). Solid grey line represents nominal significance (*p* = 1 × 10^−3^). (**B**) Gene set enrichment analysis revealed the top 10 Gene Ontology Biological Process (GO BP) terms enriched in the genes within the top 10% of dmGWAS modules.

The cell-type-specificity enriched in the genes of top 10% modules was evaluated using the bioinformatics tool WebCSEA [27] Our findings, shown in Figure 3A, indicated that monocyte was the most enriched cell type (−log_10_ raw *p*-value = 11.32), followed by muscle and glial cells (−log_10_ raw *p*-values: 8.68 and 8.67, respectively).

Overrepresentation analysis (ORA) was performed by using ClusterProfiler with consideration of Gene Ontology (GO) terms. This analysis evaluated the GO biological processes (GO-BP) of the genes included in the 10% modules. Figure 3B shows the top ten GO-BP terms enriched. Our findings showed that GO-BP terms for catabolic and metabolic processes of the amyloid precursor protein were the most enriched in the genes of the top 10% modules (adjusted *p*-value = 6.24 × 10^−6^ and 2.63 × 10^−5^, respectively). This implies that the genes within these modules are crucial in forming and degrading amyloid peptides, a pathological hallmark of AD. Notable genes within these GO terms include *ADAM10*, *BIN1*, *CLU*, and *PICALM*, all found in the top module identified by dmGWAS (Figure 2A). The visualizations of the top 10 enriched GO-CC and GO-MF terms are provided in the Supplementary Files (SF2).

The Therapeutic Target Database (TTD) provides an extended wealth of information on drugs corresponding to the investigational status of the drug, its classes, and the drug targets [28]. The genes of the top 10% of modules revealed statistically significant enrichment with 19 drug targets annotated in the TTD (*p*-value = 0.03), as determined through a hypergeometric test. Five drug targets were classified in the TTD as targets of FDA-approved medications (Table 2). Two of these FDA-approved medications (Entrectinib and Osimertinib) are indicated for neoplasms. The genes *ADAM10* and *CLU,* observed in the top dmGWAS modules, contained drug targets classified as clinical drug targets in phase 1 and phase 3 clinical trials, respectively (Aderbasib and Custirsen). These targets are currently indicated for neoplasm. The complete list of enriched drug targets is presented in the Supplementary Materials (Table S2).

### 2.4. ADAM10 Exhibits Colocalized GWAS-Methylation Quantitative Trait Loci (QTL) Signals

**Figure 4:**
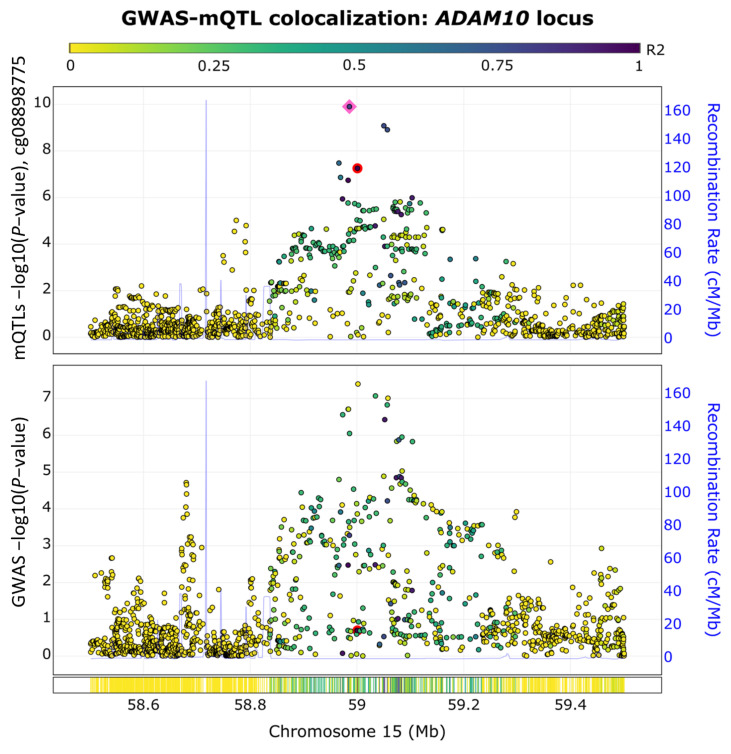
GWAS−mQTL colocalization pair in the *ADAM10* locus. The colocalization of AD GWAS signals and methylation QTL (mQTL) signals are shown for the CpG site cg08898775 at SNP rs347117 of chromosome 15. The pink square highlights the lead SNP in this locus, while the colocalized SNP is highlighted by a red dot.

To assess our enriched genetic- and epigenetic-based network, we conducted a GWAS and methylation quantitative trait loci (mQTL) colocalization analysis using the ezQTL web server [29]. We focused on the *ADAM10* gene because it is the only gene within the top module with significant genetic and epigenetic associations with AD. The ezQTL server was utilized to conduct the colocalization analysis by leveraging blood mQTL signals generated by McRae et al. [30] and utilizing the established colocalization software, eCAVIAR [29] (see Section 5). As shown in Figure 4, we observed a GWAS-mQTL colocalization pair in the *ADAM10* locus for the CpG site of cg08898775 at the SNP rs347117 (GWAS *p*-value = 4.02 × 10^−8^, QTL *p*-value = 5.58 × 10^−8^). The posterior probability for this GWAS-mQTL pair was 0.03, which surpassed the threshold of 0.01 recommended by eCAVIAR [31]. The colocalization of the AD GWAS and mQTL signals observed at this locus supported the overlap of genetic and epigenetic AD signals at the *ADAM10* locus.

## 3. Discussion

AD is a complex neurodegenerative disease characterized as the most common prevalent form of dementia; it presents a significant challenge in neurodegenerative disease research due to its complex etiology marked by the accumulation of β-amyloid and tau-neurofibrillary proteins in the brain [32]. Despite decades of research, the pathogenesis of AD remains largely elusive [32]. However, GWAS has broadened our understanding by identifying novel risk loci associated with the disease, notably highlighting the APOE gene as a critical genetic factor in disease predisposition [32,33]. This complexity necessitates the integration of genomic data with other biological data types, such as epigenetics, to advance our molecular understanding of the disease and promote novel therapeutic targets [34]. Building upon the foundation of AD, this study delved into AD’s genetic and epigenetic landscape utilizing the comprehensive GWAS dataset by Wightman et al., focusing on a cohort of European descent [23]. Our study explored the interplay between DNA methylation and genetic variations, highlighting the role of methylation—a key epigenetic mechanism—at CpG sites in the promotor region [35]. Our analysis concentrates on CpG-site signals within the TSS region to acknowledge its role in gene regulation. This specificity is crucial for understanding the complex epigenetic mechanisms influencing gene expression, in contrast to the broader genetic insights provided by whole-gene aggregation of GWAS signals [36]. Moreover, differential methylation has been identified in AD post-mortem brain samples, emphasizing the relevance of DNA methylation in the context of the disease [37]. Transitioning from this broad biological exploration, this study utilizes the dmGWAS tool [21] to integrate genetic and epigenetic datasets, providing a more granular view of AD’s molecular underpinnings.

The application of dmGWAS yielded 286 network modules with significant module scores. The top module contained nine genes, with genes *BIN1* and *GNAS* containing the highest significant GWAS node and methylation weights, respectively. The top module with the highest enrichments comprised the genes *ADAM10*, *FNBP4*, *EPHA1*, *FBXO46*, *GNAS*, *BIN1*, *CLU*, *PICALM*, and *TRIM25*. Interestingly, the *ADAM10*, *CLU*, and *PICALM* genes are directly associated with β-amyloid, a hallmark clinical point of AD [38,39]. Previous GWA studies also identified the genes *EPHA1* and *BIN1* as risk loci in AD [40,41]. *BIN1,* a gene involved with several functions, including cell cycle advance, cytoskeleton regulation, and endocytosis [42], represented the largest GWAS weight in the top module, indicating the vast variation between the SNPs of cases and control subjects within this gene region. This finding suggests that endocytosis, the process that involves the uptake and dispersion of materials within the cellular environment [43], may be dysregulated in AD cases. Indeed, dysregulation of endocytosis may lead to dysfunction in the trafficking and clearance of β-amyloid in AD [44]. The gene with the highest methylation value was *GNAS*, primarily involved in signal transduction and mediating neurotransmitters [45]. While the gene has been associated with other neurodegenerative diseases like Parkinson’s disease, there has been limited investigation for its association with AD [46]. The gene *TRIM25*, whose function is primarily involved in immune response [47], is the central node, interacting with most genes in the network. Therefore, while *TRIM25* may not have any naïve association with AD, it is an important interactor with several genes associated with AD.

We witnessed the integration of methylation data and the genetic variation of *ADAM10*, a gene associated with AD for its role in the processing of the amyloid precursor protein (APP), wherein it cleaves APP within the β-amyloid region [39]. As β-amyloid buildup is a clinical indicator of AD, this could indicate that decreased expression of this gene is related to the lack of β-amyloid clearance, resulting in the development of the disease [48]. Our GWAS and mQTL colocalization analysis of *ADAM10* highlights a crucial overlap of genetic and epigenetic signals in AD, reinforcing our understanding of its molecular underpinnings. The colocalization findings suggest that genetic and epigenetic modifications contribute to *ADAM10′s* regulatory effects as an α-secretase, which emphasizes its role in the non-amyloidogenic pathway [49]. *ADAM10* was not indicated to have an FDA-approved target. However, our findings identified the drug Aderbasib, currently being investigated in a clinical trial (NCT04295759), for its potential benefits in treating children with recurrent and progressive high-grade gliomas. While studies have explored the antineoplastic activity of Aderbasib in many cancer types [50,51,52], to our knowledge, the drug has not been studied in the context of AD treatment. These findings suggest that *ADAM10* is a valuable biomarker for investigating AD risk [12] and supports its potential as a therapeutic target, offering novel avenues for AD treatment and diagnosis strategies [53,54].

To explore their biological significance, we performed cell type and gene set enrichment analysis in the genes (n = 74) of the top 10% of modules. We provided results from the top 20 major cell types reported from our WebCSEA analysis [27]. The WebCSEA results denoted monocytes, muscle cells, and macrophages as the most enriched major cell types. Our analysis indicates that the genes reported are enriched in immune function-related cell types. It has been hypothesized that increased levels of inflammatory cytokines in the central nervous system correlate with AD progression [55]. The observed significant enrichments in monocytes and macrophages may be surrogate indicators of this phenomenon.

An over-representation analysis of the genes in the top 10% of dmGWAS modules revealed the enrichment of GO BP terms related to amyloid-beta formation through regulating APP metabolism and catabolic developments (Figure 3B). This indicates that genes enriched within the top 10% of modules may reflect the dysregulation of these processes, which are hallmarks of AD pathology. Furthermore, the molecular functions identified peptide and amyloid-beta binding (SF2), which underscores their crucial role in AD pathogenesis and reinforces the relevance of the findings in this study [56].

Given the recent decades’ lack of progress in AD therapeutics, there is a pressing need to uncover repurposable drug targets to prompt more effective treatment options [57]. The drug target analysis indicated that five targets of FDA-approved drugs were present within our top 10% of modules: *APP, CRHR1, EGFR, ESR2,* and *NTRK1* (Table 2). While the target gene *APP* is identified as a target of Florbetapir F-18, this drug’s primary use currently lies in diagnostic imaging, specifically as a radiopharmaceutical compound in PET scans to visualize amyloid plaques, a key indicator of Alzheimer’s Disease [58]. A subsequent drug target identified by our analysis was *CRHR1*, whose function includes stress response by facilitating the effect of corticotropin-releasing hormones. Previous studies have shown that it may play a role in AD by modulating stress-related neuroinflammation [59]. The corresponding drug, Telavancin, is an antibiotic utilized to treat Staphylococcus infection by disrupting the bacterial membrane [60]. To our knowledge, this drug has yet to be recognized as a potential therapeutic for AD. However, research in AD mouse models has investigated how antibiotic-induced alterations in gut microbiome diversity can impact neuro-inflammation and amyloid accumulation [61]. This presents a novel area for exploration within our treatment strategies regarding AD. Another target in our top modules was *EGFR*, whose overexpression and mutations are well explored in the cancer domain, with the primary function including the initiation of tyrosine kinase signaling cascades [62]. Our findings highlighted Osimertinib, a tyrosine kinase inhibitor typically used to treat non-small cell lung cancers. It is particularly relevant to AD due to its permeability across the blood–brain barrier, bypassing a hurdle for AD therapeutic options [63]. A previous study conducted by Advani et al. investigated the potential therapeutic targeting of anticancer drugs in AD by performing an in silico multi-omic analysis [64]. Our study also identified estrogen receptor beta (*ESR2*) as statistically significant in our GWAS and methylation Z-scores. Polymorphism within *ESR2* has been investigated for association with AD development in women [65]. Furthermore, a study conducted by Saleh et al. suggested increased cognitive advantages in APOE-ε4 carrier women taking estrogen conjugates in hormone replacement therapy (HTR) when compared to non-HRT participants [66]. However, subsequent research is needed to validate these findings. The neurotrophic tyrosine kinase receptor 1 (*NTRK1)* target regulates neuronal development, and its dysregulation is associated with cognitive disabilities and neuronal damage [67]. This gene was significantly hypermethylated in our network analysis. Our analysis identified that Entrectinib, a drug utilized in cancer treatment as a tyrosine kinase inhibitor, has the potential to be repurposed in AD, as it affects the neuroinflammation pathways implicated in AD pathology. Utilizing the TTD to pinpoint these targets, our findings demonstrate how the database can play a crucial role in supporting drug repositioning by revealing how FDA-approved drugs can be explored in AD due to their molecular targets identified in the preceding analysis [28]. Previously, Li et al. have identified this drug for its potential in AD treatment through transcriptomic weighted gene co-expression network analysis data [68]. Therefore, the biological relevance of our drug target analysis provides a systematic approach for classifying targets based on their drugability characteristics, thus indicating several potential therapeutic candidates for novel AD treatment [69].

In the current study, dmGWAS was utilized as a multi-omics approach to derive insights into AD; however, subsequent studies might benefit from using the edge-weighted dmGWAS (EW_dmGWAS) tool [70]. EW_dmGWAS is an advanced iteration of the dmGWAS tool, which can integrate gene expression data by applying weighted edges of differential gene co-expression, offering a refined exploration of disease-related genetic and epigenetic interplays. Liu et al. conducted an integrative proteomics and GWAS study to investigate the molecular pathways in brain-specific regions to recognize causal genes in AD pathogenies [71]. While the current study focused on the epigenetic mechanisms occurring in AD, succeeding studies integrating genetic, proteomic, and epigenomic data may provide a more holistic view of AD’s molecular landscape.

Limitations within the current study must be acknowledged when interpreting the findings presented in this study. First, the integrated data sources present limitations, given that the GWAS summary statistics and the DNA methylation data were derived from different cohorts. Although matching participant samples could yield more insightful findings, practical challenges related to scale, cost, and duration pose substantial constraints. The second limitation is observed in the methods used to transform the epigenetic data into gene-level methylation scores. Although this method was applied in a previous study [22], it does not consider many differentially methylated CpG sites outside the promoter region. Future studies may expand these methods to incorporate other differentially methylated regions in network analysis. A third limitation can be observed in our analysis which leverages established PPI networks. The PPI inherently focuses on previously identified networks and structures, limiting the discovery of novel interactions. Moreover, a bias might be postulated as more well-studied genes may have extensive literature and biological data, reflected in the PPI connectivity (i.e., hub genes) and the subsequent gene enrichment analysis. However, the value of this approach lies in the high-quality curation of the PPI, which offers reliable and biologically relevant findings. These limitations highlight areas of consideration for future studies.

## 4. Conclusions

Due to the multifactorial complexity of AD, this study aimed to integrate genetic risk factors identified from large-scale GWAS data and epigenetic signals from DNA methylation through a network module approach. Furthermore, our findings indicated *TRIM25* as the central node in our top module and an important interactor in AD. *TRIM25* was connected to several highly significant GWAS signal genes despite lacking GWAS and methylation signals. Furthermore, the gene *GNAS* in our top module had the largest methylation signal, indicating hypermethylation. Subsequent enrichment analyses of genes within our top 10% of modules suggested that our gene networks were relevant to known functions of AD pathology. Additionally, our findings identified the FDA-approved therapeutic drug targets *EGFR*, *ESR2,* and *NTRK1* for potential drug repurposing of AD treatment. *ADAM10* was indicated in several analyses, exhibiting synergistic genetic and epigenetic signals. This proposes ADAM10 as an important gene in AD regulatory mechanisms and presents an unexplored target for novel therapeutic treatments in the disease. Therefore, by combining genetic and epigenetic data, this integrative network approach improved our understanding of AD’s etiology and supported additional avenues for targeted therapeutic interventions, potentially leading to more effective treatment strategies for AD.

## 5. Materials and Methods

### 5.1. Collection of AD-Specific Multi-Omics Data

The AD GWAS summary statistics data included genome-wide SNP-level associations for AD cases leveraging the GWA study by Wightman et al. [23]. This study is the largest source of LOAD GWAS data, comprising 39,918 cases and 358,140 controls, totaling 398,058, after excluding participants from the UK Biobank (UKB), 23andMe, and proxy cases.

The DNA methylation data used in our study originated from the Religious Orders Study and Memory and Aging Project (ROSMAP). This organization provides a longitudinal study of AD and related neurodegenerative conditions [24]. This study investigated biomarkers related to cognitive decay and AD developmental risk. The methylation data comprised samples from 549 individuals, 324 cases, and 225 control participants. Tissue samples for methylation data were sourced from post-mortem dorsolateral prefrontal cortex brain tissue. Post-mortem AD diagnosis was performed leveraging guidelines by the NIA-Reagan criteria and modified CERAD [24]. Additionally, neurofibrillary pathology assessment through Braak Staging was utilized [24]. The Illumina HumanMethylation450 BeadChip acquired CpG-level methylation intensities.

The reference network for human PPIs was retrieved from the BioGRID database. This dataset contained a collection of experientially validated PPI annotations, and it was publicly available and accessible through the BioGRID database [25].

### 5.2. Pre-Processing of GWAS and DNA Methylation Data

The integration of genetic and epigenetic data via dmGWAS necessitated the input of gene-level weights. GWAS-based gene-level *p*-values were obtained utilizing the gene analysis command line toolkit MAGMA (Multi-marker Analysis of Genomic Annotation) [72]. First, SNP-to-gene annotation was performed utilizing a 35 kb upstream and 10 kb downstream window around gene loci. Subsequently, gene-level analysis was performed using an SNP-wise mean model within MAGMA. This approach adopts a multiple regression model to address linkage disequilibrium (LD) and elucidate the cumulative effects of genetic variants in each gene region, thereby integrating aggregating the effects of SNPs within the annotated genes, yielding gene-level GWAS *p*-values [72]. We transformed these gene-level *p*-values to GWAS Z scores via the inverse normal distribution function.

The R statistical programming language was used to pre-process the GWAS summary statistics and the DNA methylation data [73]. Due to numerous variants with significant association and extensive linkage disequilibrium in the *APOE* gene region, Ware et al. removed this gene locus (chr19: 45,384,477–45,432,606 bp) [74]. In this study, we specifically removed the SNPs from the following locus: chr19: 44,000,000–46,000,000 bp.

The process of converting epigenetic data into gene-level scores required managing the methylation intensities obtained from CpG-level probes. This measurement is observed in DNA methylation analysis, as CpG sites are the primary genomic sites for methylation [75]. The Limma R package was employed to process the methylation data and obtain the association scores for differentially methylated CpG-level sites [76].

Stouffer’s Z-score method assisted in adjusting the additive effect of methylation intensities at the transcription start site (TSS) regions of genes [22,77]. To apply this, we first annotated the CpGs to the TSS of genes. The genomic coordinates of the CpG sites were mapped to the TSS regions of the candidate genes utilizing annotations defined in the Illumina Human Methylation 450 BeadChip annotation file. The TSS of the genes captures up to 1500 base pairs upstream of the TSS, thereby incorporating the promoter region of the gene influential in gene regulation. The Stouffer’s Z-score method was adapted to obtain gene-level methylation scores using the following Equation (1):

(1) $$Z_{CpG}=sign\left({Log}_{2}FC\right)\times \varphi^{-1}\left(\frac{p}{2}\right),Z_{methy}=\frac{\sum_{i=1}^{k}Z_{CpG}}{\sqrt{k}}$$

Here, we denote $Z_{CpG}$ as the Z-score derived from the association *p*-value of a CpG probe, and $Z_{methy}$ is the methylation score for a specific gene. $Z_{CpG}$ was acquired through the inverse distribution function, denoted by $\varphi^{-1}$. The *sign* ${Log}_{2}FC$ component is the positive or negative fold change, *p* is the *p*-value associated with the *CpG* probe, and *k* is the number of *CpG*s annotated for a specific gene. This method allowed us to detail the directionality of methylation. In this way, a positive Z-score indicated that hypermethylation was observed in the TSS region, suggesting the downregulation of gene expression. Conversely, a negative Z-score indicated hypomethylation, suggesting upregulation of gene expression. The R script “Aggregate_TSS_CpGs.R” derives the gene-level methylation scores (Figure 1).

### 5.3. Integration of GWAS and DNA Methylation Data Utilizing dmGWAS

As illustrated in Figure 1, MAGMA was utilized to derive gene-level GWAS Z scores, and the Aggregate_TSS_CpGs.R script was utilized to derive gene-level methylation Z scores. Next, we aggregated our genetic and epigenetic weights to incorporate them into dmGWAS. To do this, we incorporated a scaling factor to minimize variance during data integration, using the ratio of variances. The scaling factor was the variance observed in the GWAS Z scores divided by the variance observed in the DNA methylation Z scores.

### 5.4. Dense Module Searching Genome-Wide Association Studies Signals via dmGWAS

Our in-house dmGWAS tool incorporates association signals from GWAS summary statistics into a given PPI network to locate subnetworks of genes via the dense module searching algorithm. The dmGWAS algorithm is a greedy search algorithm and a statistical method that identifies local maximum proportions of gene node weights, as described by Jia et al. [21]. The local maximum is recognized by calculating module scores (*Z_m_*), wherein the sum of node weights is divided by the square root of the number of nodes in each module. The equation for the calculation of *Z_m_* is the following:

(2) $$Z_{m}=\frac{\sum Z_{i}}{\sqrt{k}}$$

In Equation (2), $Z_{i}$ represents node weights for each gene, and k denotes the number of genes in each module. In our study, *Z_i_* represented the aggregated genetic and epigenetic *Z* scores obtained from the GWAS and methylation data. The module scores determine whether or not genes in each module are associated with the disease. Visualization of the networks was achieved using the open-source software Cytoscape version 3.9.1 [26]. The Cytoscape interface allows for various visualization methods to represent data.

### 5.5. Cell Type and Gene Set Enrichment Analyses

Functional enrichment analyses were performed to interpret the findings of our AD gene networks. The input data for our functional enrichment analysis were a gene list of the top 10% dmGWAS modules with the highest module scores. This gene list was comprised of a total of 74 genes. Web-based Cell-type-Specific Enrichment Analysis (WebCSEA), an online bioinformatics tool developed by members of our team, was utilized to recognize cell-type specific gene expression patterns from transcriptomic data (https://bioinfo.uth.edu/webcsea/, (accessed on 13 July 2023)) [27]. These genes were then mapped to a reference gene set for functional annotation. Two levels of statistical significance were considered, as shown in Figure 3A. The grey line denoted the nominal significance (*p* = 1 × 10^−3^). The red dashed lines denoted the Bonferroni-corrected significance (*p* = 3.69 × 10^−5^).

Additionally, we conducted an overrepresentation analysis (ORA) of Gene Ontology (GO) terms using the Cluster Profiler R package [78]. Our analysis utilized the human-specific annotation database “org.Hs.eg.db”. This analysis was subdivided into the terms GO Biological Process (GO-BP), GO Cellular Component (GO-CC), and GO Molecular Function (GO-MF). The adjusted *p*-values were calculated using the Benjamin–Hochberg procedure to account for the false discovery rate. The Cluster Profiler R Package was also utilized to visualize the top 10 GO-BP terms (Figure 3B). Other enriched terms in the GO-CC and GO-MF categories are displayed in SF2.

### 5.6. Drug Target Analysis

The Therapeutic Target Database (TTD) provides extensive information about therapeutic molecular targets, associated diseases, and drugs. Moreover, the database contains knowledge corresponding to the investigational status of the drugs [28]. Drug target enrichment analysis was performed utilizing our in-house TargetEnrich.R script. Data concerning drug targets and associated drug information were extracted from the TTD websites. Subsequently, a hypergeometric test was performed to calculate the level of enrichment. This test considered 2578 drug target genes derived from TTD, 19 drug target genes in our network, 74 genes within the top 10% modules, and 15,114 genes used as input in the dmGWAS process.

### 5.7. GWAS-mQTL Colocalization Analysis

We performed a colocalization analysis of the AD GWAS signals with mQTL (methylation quantitative trait loci) data to further assess the overlapping genetic and epigenetic signals. Specifically, we utilized the ezQTL server, which facilitated the analysis by providing a preprocessed mQTL dataset. We utilized the ‘Whole_Blood_McRae’ mQTL association data generated by McRae and colleagues, incorporating 52,916 cis mQTLs and 2025 trans mQTLs from 1366 individuals using Illumina HumanMethylation450 arrays [30]. Other specifications for applying ezQTL included AD GWAS summary statistics of the specific locus, linkage disequilibrium information, and specific locus information. Our analysis specified the cis-QTL distance within the ±500 kilobase pair region of the rs347117 variant, situated on chromosome 15 at position 59000957. Additionally, we indicated the LD panel from the 1000 Genomes Project European population. To determine significant thresholds for GWAS-mQTL colocalization, we referred to original threshold recommendations by authors of the eCAVIAR software. Thus, we used a posterior probability (PP) threshold of 0.01 for the significance of the results of eCAVIAR [31].

## Figures and Tables

**Table 1:** Description of datasets integrated into dmGWAS analysis [23].

Dataset	Data Type	Sample Size
Wightman et al.	GWAS	N = 398,058 (Case = 39,918, Control = 358,140)
ROSMAP	DNA methylation	N = 549 (Case = 325, Control = 224)
BioGRID: reference network	Protein–protein interaction	N = 536,844 edges M = 19,087 proteins

**Table 2:** Summary of FDA-approved drug targets from the TTD enriched in top 10% modules.

Target Gene	Target Name	Drug Compound	Indication
*APP*	Amyloid beta A4 protein	Florbetapir F-18	Diagnostic Imaging [ICD-11: N.A.]]
*CRHR1*	Corticotropin-releasing factor receptor 1	Telavancin	Staphylococcus infection [ICD-11: 1B5Y]
*EGFR*	Epidermal growth factor receptor	Osimertinib	Non-small-cell lung cancer [ICD-11: 2C25.Y, ICD-9: 162]
*ESR2*	Estrogen receptor beta	Conjugated Estrogens	Menopause symptom [ICD-11: GA30.0, ICD-9: 627.2] Vasomotor symptom [ICD-11: CA08, ICD-10: J30-J39, J30, ICD-9: 627.2]
*NTRK1*	Tropomyosin-related kinase A	Entrectinib	Non-small cell lung cancer [ICD-11: 2C25] Neuroblastoma [ICD-11: 2D11.2] (Phase 1)

## Data Availability

This research is based on datasets available in online repositories. The DNA methylation data of AD and controls supporting this study’s findings are available from the Synapse portal (https://www.synapse.org/#!Synapse:syn3157275, (accessed on 12 December 2022). All in-house R scripts utilized in our workflow are readily accessible and openly available through our GitHub repository, which can be accessed at: https://github.com/astrika/Analytical-Approach-for-MS-GRN, (accessed on 2 February 2023).

## Supplementary Materials

The following supporting information can be downloaded at: https://www.mdpi.com/article/10.3390/epigenomes8020014/s1; Figure S1: A complete distribution of gene level GWAS and methylation weights; Figure S2: Top 10 over-representation analysis of GO-CC and GO-MF terms; Table S1: Complete list of node weights; Table S2: Complete list of drug targets identified by TTD drug target analysis.
