# Supplementary figures and images for: Unveiling Gene Interactions in Alzheimer’s Disease by Integrating Genetic and Epigenetic Data with a Network-Based Approach
Source: Epigenomes. 2024 Apr 1;8(2):14. doi: 10.3390/epigenomes8020014 (PMC11036294; doi:10.3390/epigenomes8020014)

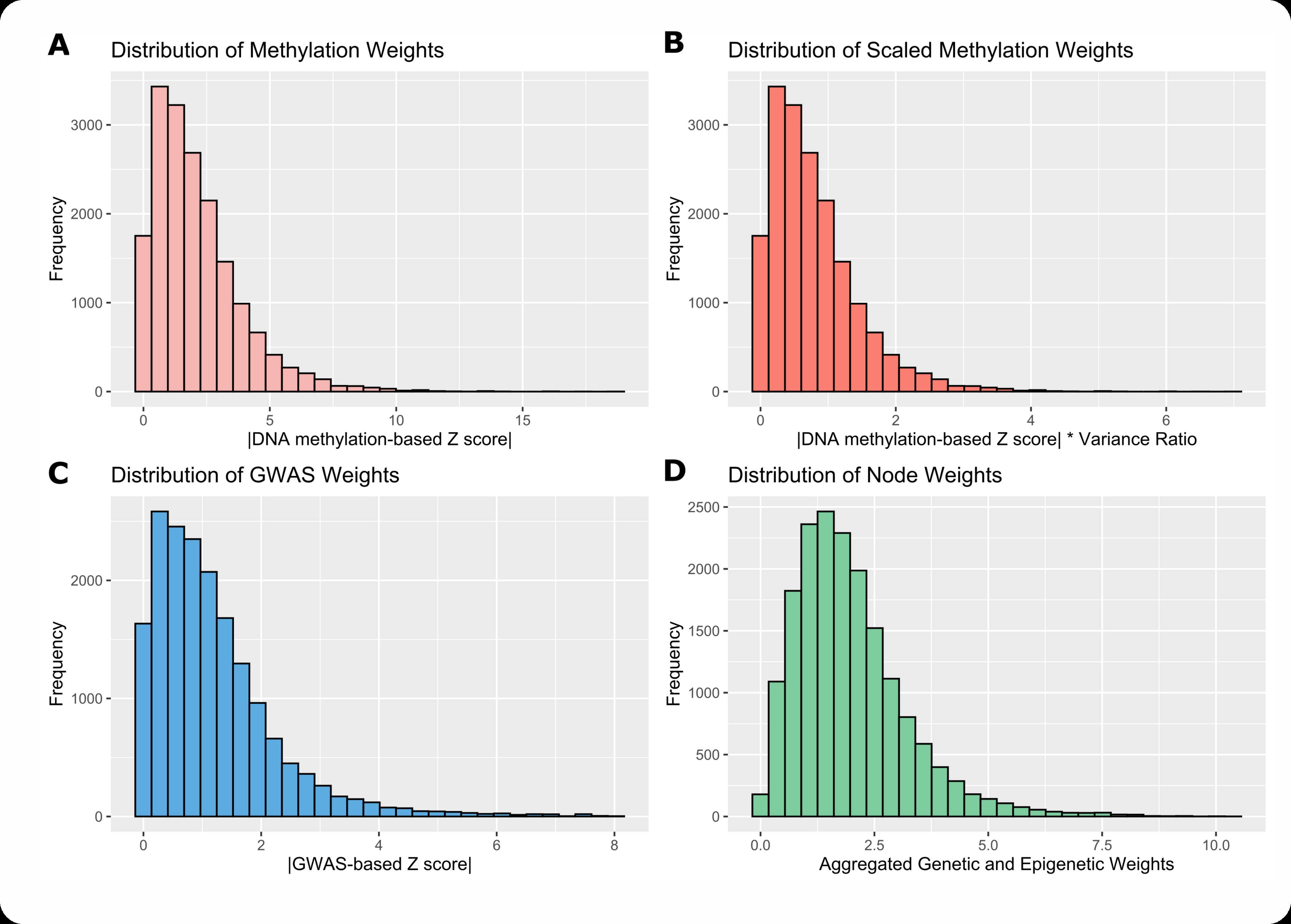

Supplement: Supplementary file 1 [file epigenomes-08-00014-s001.zip › Figure_S1.jpeg]

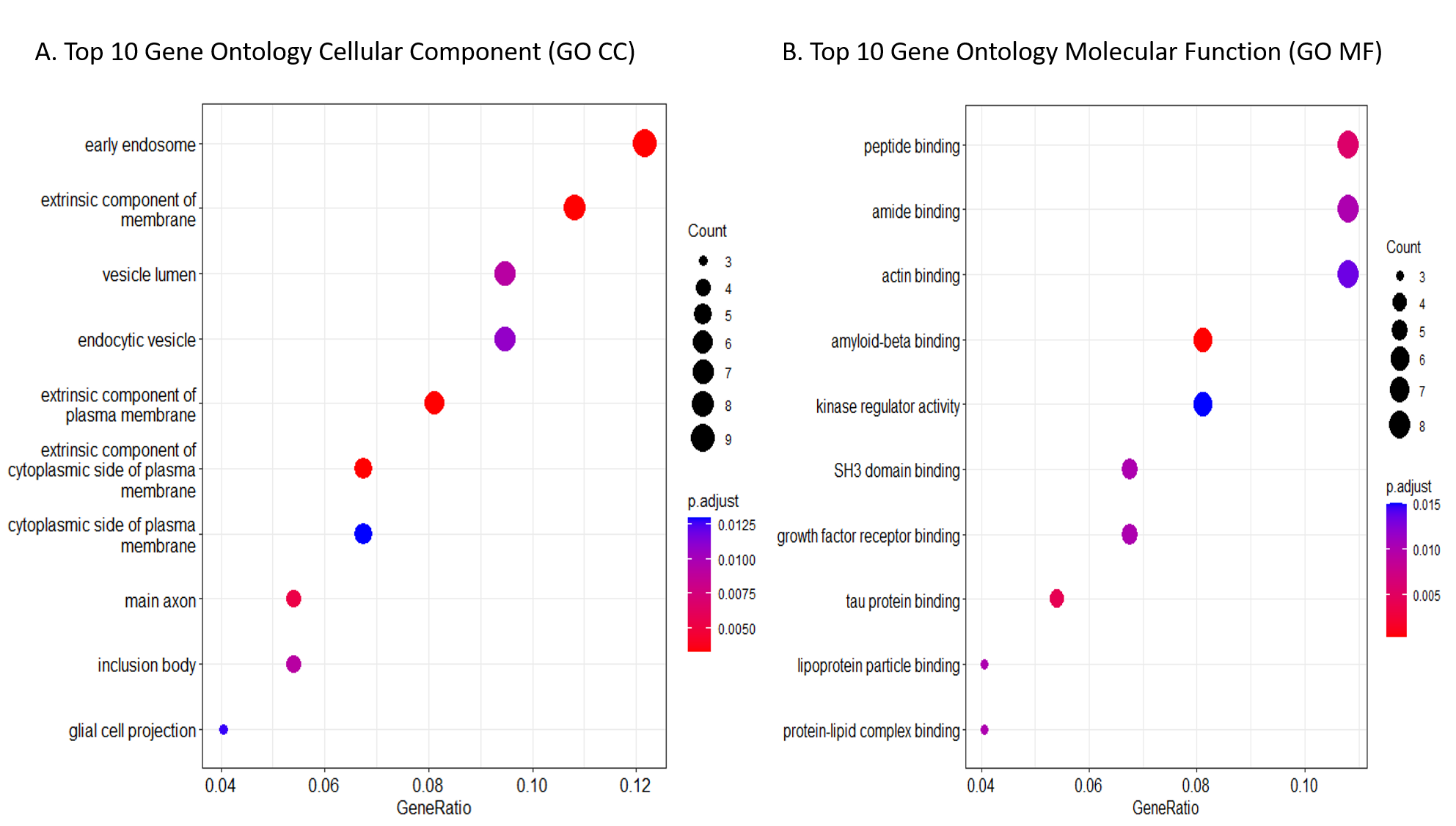

Supplement: Supplementary file 1 [file epigenomes-08-00014-s001.zip › Figure_S2.png]
